# A role for the p53 tumour suppressor in regulating the balance between homologous recombination and non-homologous end joining

**DOI:** 10.1098/rsob.160225

**Published:** 2016-09-21

**Authors:** Sylvie Moureau, Janna Luessing, Emma Christina Harte, Muriel Voisin, Noel Francis Lowndes

**Affiliations:** Genome Stability Laboratory, Centre for Chromosome Biology and School of Natural Science, Biomedical Science Building, National University of Ireland Galway, Dangan, Ireland

**Keywords:** p53, 53BP1, BRCA1, non-homologous end joining, homologous recombination r, DNA double-strand break repair

## Abstract

Loss of p53, a transcription factor activated by cellular stress, is a frequent event in cancer. The role of p53 in tumour suppression is largely attributed to cell fate decisions. Here, we provide evidence supporting a novel role for p53 in the regulation of DNA double-strand break (DSB) repair pathway choice. 53BP1, another tumour suppressor, was initially identified as p53 Binding Protein 1, and has been shown to inhibit DNA end resection, thereby stimulating non-homologous end joining (NHEJ). Yet another tumour suppressor, BRCA1, reciprocally promotes end resection and homologous recombination (HR). Here, we show that in both human and mouse cells, the absence of p53 results in impaired 53BP1 focal recruitment to sites of DNA damage induced by ionizing radiation. This effect is largely independent of cell cycle phase and the extent of DNA damage. In p53-deficient cells, diminished localization of 53BP1 is accompanied by a reciprocal increase in BRCA1 recruitment to DSBs. Consistent with these findings, we demonstrate that DSB repair via NHEJ is abrogated, while repair via homology-directed repair (HDR) is stimulated. Overall, we propose that in addition to its role as an ‘effector’ protein in the DNA damage response, p53 plays a role in the regulation of DSB repair pathway choice.

## 1. Introduction

The p53 transcription factor is crucial for the maintenance of genome integrity [1,2]. Its role in tumour suppression has been largely associated with cell fate decisions upon damage with the potential to eliminate cancerous cells without affecting organismal integrity. Immediately after DNA damage, p53 regulates transient delays to cell cycle progression believed to allow cells greater time to repair genome damage prior to key cell cycle transitions, especially the transit from G1 into S phase. In the case of substantial DNA damage, p53 can regulate permanent exit from cell proliferation via either senescent or apoptotic mechanisms [2]. Interestingly, in the absence of crucial p53 target genes required for regulating the G1/S checkpoint, apoptosis and senescence, p53 retains some tumour suppressive functions, including genome stability [3], suggesting at least one further role for p53 in regulating tumour suppression. A candidate role could be direct regulation of DNA repair.

Cells have developed various strategies to respond to the many types of DNA damage [4]. Base excision repair (BER), nucleotide excision repair (NER), mismatch repair (MMR) and trans-lesion synthesis (TLS) are the four major pathways processing lesions affecting only one strand of the DNA [4,5]. The p53 protein has been implicated in all four of these pathways either through its role as a transcription factor of genes required for efficient single-strand break repair or through direct protein–protein interaction with repair factors. It is worth noting that the role of p53 in BER could be cell-cycle-specific as it has been reported to enhance BER in G0 and G1, while being inhibitory in G2 and M phase [6]. With respect to DNA double-strand breaks (DSBs), p53 was shown in 1994 to interact with 53BP1, a DSB repair factor (discussed below), which interacts with the DNA binding domain of p53 through its BRCT domain and is reported to enhance p53 transcriptional activity [7–10].

DSBs are the most challenging and potentially harmful DNA lesions that a cell can encounter as genetic information can be altered through deletion, mutation or rearrangement. To repair these breaks cells have developed two principal repair mechanisms, one of which requires a homologous template and is termed homology-directed repair (HDR), also known as homologous recombination (HR) [11], while the other is homology independent and termed non-homologous end joining (NHEJ), or illegitimate recombination [12]. NHEJ-dependent repair catalyses the re-ligation of the broken DNA ends, sometimes with loss of one or more nucleotides resulting in error-prone repair. HDR occurs mainly during S and G2 when an intact sister chromatid is easily available as the preferred homologous template. In HDR, break detection is followed by one strand at each end being resected in the 5′ to 3′ direction resulting in a 3′ single-stranded DNA overhang that is initially coated by RPA and subsequently by RAD51. RAD51 is a recombinase that catalyses the search for intact homologous DNA sequences and subsequent strand exchange.

Conflicting results have been obtained when assessing the role of p53 in either HR or NHEJ. Using an episomal plasmid-based re-joining assay in mouse embryonic fibroblasts (MEFs), enhanced DNA end joining of short complementary ends in the presence of p53 has been reported [13], suggesting a role for p53 in the promotion of NHEJ. This *in vivo* role was supported by enhanced *in vitro* re-ligation of linearized plasmids in cellular extracts from p53 defective cells [14]. However, p53 has also been reported to downregulate NHEJ. For example, reduced NHEJ-dependent repair of I-*Sce1*-induced DSBs has been reported in the presence of p53 [15].

Involvement of p53 in HR is also subject to controversy. It has been suggested that p53 could suppress HR-dependent repair either through transcriptional repression of the HR factors RAD51 and BRCA1 or through direct protein–protein interactions with RPA, RAD51 and the RecQ helicases [6,16,17]. However, other studies did not observe any defect in the HR pathway in absence of p53 [18,19]. Recently, a model has been proposed suggesting crosstalk between the HR and NHEJ pathways regulated via phosphorylation of a p53-RPA complex by the PIKK kinases: ATR, ATM and DNA-PK [20]. These authors suggested that a low level of p53 is associated with RPA under non-stressed conditions, while upon DNA damage RPA is phosphorylated by DNA-PK and p53 is phosphorylated in an ATR-ATM-dependent manner. This resulting dissociation of the RPA-p53 complex was speculated to allow each protein to perform their respective functions in both DNA repair and cell cycle regulation.

The balance between DSB repair via either the HDR or NHEJ pathways has also been reported to be regulated via the DDR mediator proteins 53BP1 and BRCA1 [21–25]. While 53BP1 at DSBs inhibits DNA resection, thereby preventing HDR-dependent repair, BRCA1 recruitment to DSBs enhances the resection required for HDR [26,27]. The significance of reduced resection and consequently reduced HDR is that the increased genomic instability and cancer predisposition observed in *Brca1* knockout mice can be suppressed by co-deletion of *53Bp1* [21,28].

The rapid relocation of 53BP1 and BRCA1 to DSBs is easily monitored after ionizing radiation by the appearance of so-called ionizing radiation-induced foci (IRIF) within the nuclei of cells. Upon DNA damage, the histone variant H2AX is phosphorylated at serine 139. MDC1 binds directly to γH2AX and facilitates the recruitment of numerous components of the DNA damage response (DDR) including the E3-ubiquitin ligases, RNF8 and RNF168. Mono- and poly-ubiquitination of H2A-type histones in the vicinity of the DSB facilitate the recruitment and/or retention of 53BP1 and BRCA1-containing complexes [29–32]. Interestingly, 53BP1 recruitment requires the dynamic binding of its tandem Tudor domain with dimethylated histone H4 (H4K20me2), while its stable retention at chromatin surrounding DSBs requires a newly described ubiquitin-binding domain and RNF8/RNF168-dependent ubiquitination [33]. Lack of H4K20me2 has been reported to result in nearly complete abrogation of 53BP1 foci formation in HeLa cells for at least an hour after DNA damage induction [34–36]. In contrast, another study in MEFs has shown that lack of H4K20me2 results in a partial defect of 53BP1 IRIF exclusively during the first 5 min after DNA damage [37]. However, the different p53 status of the cell lines under investigation was not considered. In this respect, it is intriguing that other studies provide evidence for accumulation of p53 at sites of DNA damage—specifically, a form of p53 that is dimethylated on lysine 382 (p53K382me2) after DNA damage [38,39]. Furthermore, p53K382me2 was reported to have increased affinity for the tandem Tudor domain of 53BP1 [38,40].

Here, using human and primary mouse cell lines, we demonstrate that p53 regulates the recruitment of 53BP1 to sites of DSBs. In the absence of p53, recruitment of 53BP1 is less efficient, especially in G1 and early S phase, while recruitment of BRCA1 to DSBs is reciprocally promoted by lack of p53. Consistent with these results, recruitment of the RAD51 recombinase to sites of DSBs is also increased while recruitment of MDC1, which functions upstream of both BRCA1 and 53BP1, is not affected. We provide further support for the enhanced HDR implied by increased RAD51 recruitment to DSBs in p53-defective cells and through monitoring DSB repair in cells treated with specific topoisomerase inhibitors. Furthermore, we show decreased sensitivity to PARP inhibitors and increased rates of HDR in p53-depleted cells. Our study highlights a regulatory role for p53 early in the DDR in the regulation of the appropriate balance between competing DSB repair pathways. Specifically, we suggest that p53 is required for fine-tuning the balance between the recruitment of competing tumour suppressors, 53BP1 and BRCA1, to DSBs.

## 2. Results

### 2.1. Efficient recruitment of 53BP1 into ionizing radiation-induced foci requires p53

**Figure 1.**
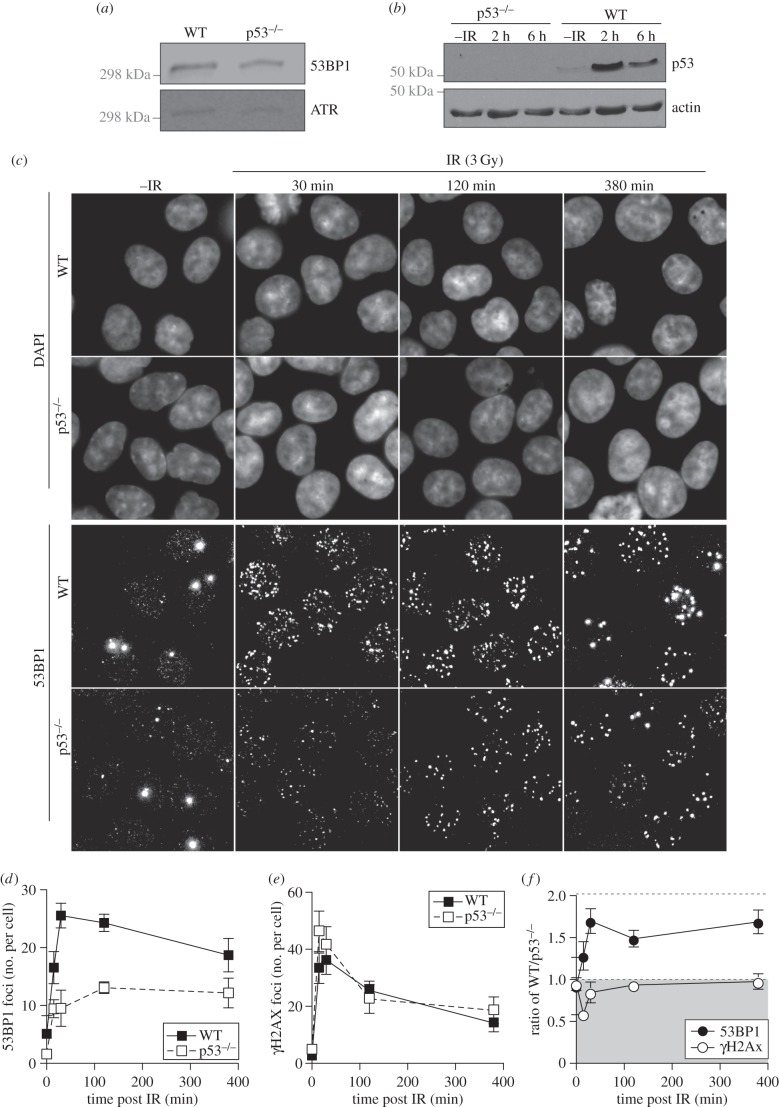
p53 promotes 53BP1 recruitment to DNA damage sites in human HCT116 cells. (*a*) 53BP1 protein levels in whole cell extracts prepared from either WT or p53-null HCT116 cells were analysed by western blotting. (*b*) p53 protein level in whole cell extracts from WT and p53-null HCT116 cells either before or after IR exposure (3 Gy) were analysed by western blotting. (*c*) Detection of endogenous 53BP1 by immunofluorescence in WT and p53-null HCT116 cells. Cells were either mock treated or irradiated with 3 Gy and allowed to recover for the indicated times before being fixed, stained with 53BP1 antibody and visualized on a Deltavision microscope. Note that large bright foci in unirradiated cells are 53BP1 nuclear bodies. (*d*) Quantification of the number of 53BP1 foci. (*e*) Quantification of the number of γH2AX foci. (*f*) Ratio of 53BP1 and γH2AX focal intensity in WT cells relative to p53-null HCT116 cells.

The Tudor domain of 53BP1, required for 53BP1 recruitment to DSBs, has also been reported to bind to a dimethylated lysine on the C-terminal of p53 (p53K382me2), suggesting a role for p53 at DSBs [38,40]. To assess whether p53 could regulate the recruitment of 53BP1 to DSBs, we assayed 53BP1 ionizing radiation-induced foci (IRIF) formation in human HCT116 WT and isogenic p53-null cells [1]. While expression of 53BP1 is normal in these p53-null cells, p53 cannot be detected either before or after IR (figure 1*a*,*b*). We detected significantly fewer and less intense 53BP1 IRIF in p53-null cells compared with the WT, whereas γH2AX foci were not affected by loss of p53 (figure 1*c*; electronic supplementary material, figure S1). Quantification of the number of foci per cell revealed fewer detectable 53BP1 IRIF in *TP53*^−/−^ HCT116 cells (figure 1*d*), whereas the number of γH2AX IRIF was not significantly different between either cell line (figure 1*e*). This defect in 53BP1 IRIF could also be detected by quantifying focal intensity (figure 1*f*). The ratio of average focal intensity per cell between the two cell types revealed that 53BP1 IRIF were significantly brighter relative to p53-null cells across the time course used, whereas γH2AX IRIF were not more intense in WT relative to p53-null cells.

**Figure 2.**
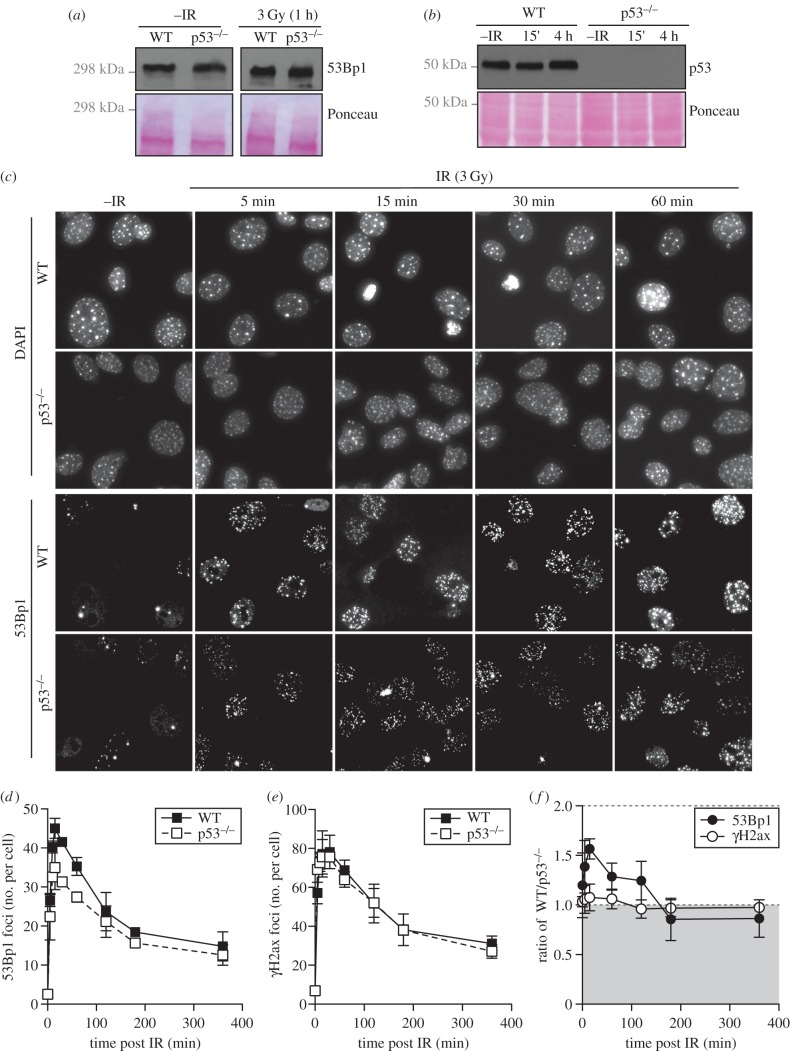
p53 promotes 53Bp1 recruitment to DNA damage sites in MEFs. (*a*) Mouse 53Bp1 protein levels in whole cell extracts prepared from WT or p53-null MEF cells either before or after IR exposure (3 Gy) were analysed by western blotting. (*b*) p53 protein levels in whole cell extracts from WT and p53-null MEF cells either before or after IR exposure (3 Gy) were analysed by western blotting. Note that in early passage MEFs, but not later passage nor transformed MEFs, high levels of p53 are detected irrespective of damage [41]. (*c*) Detection of endogenous mouse 53Bp1 by immunofluorescence in WT and p53-null MEFs. Cells were irradiated with 3 Gy, fixed at the indicated time and stained with 53Bp1 antibody. Note that the focal structures detected in MEFs by DAPI staining are heterochromatic foci. (*d*) Quantification of the number of 53BP1 foci. (*e*) Quantification of the number of γH2AX foci. (*f*) Ratio of γH2AX and 53Bp1 focal intensity in WT cells relative to p53-null MEFs.

We also examined the recruitment of mouse 53Bp1 into IRIF using early passage MEFs (either WT or null) for p53 (figure 2). Mouse 53Bp1 is expressed normally in these *TRP53^−/−^* MEFs both before and after irradiation (figure 2*a*), whereas p53 could not be detected in p53-null cells, as expected (figure 2*b*). Similarly, to our results in human HCT116 cells, early passage MEFs also displayed deficient IRIF formation of mouse 53Bp1 in p53-null relative to WT cells (figure 2*c*). In MEFs, the number of detectable 53Bp1 IRIF is significantly reduced in p53-deficient MEFs relative to WT within the first 2 h after IR (figure 2*c*,*d*), whereas γH2AX foci remained unaffected by p53 status (figure 2*e*; electronic supplementary material, figure S2). In addition to reduced 53Bp1 focal number, 53Bp1 focal intensity was weaker in MEFs deficient for p53 compared with WT cells in the first 2 h after IR (figure 2*f*). As 53BP1 expression is unaffected by the status of the p53 transcription factor (figures 1*a* and 2*a*) our data are consistent with a role for p53 in the efficient recruitment of 53BP1/53Bp1 to sites of DNA damage in both human and mouse cell types.

### 2.2. p53 is required for efficient 53BP1 IRIF formation irrespective of the extent of DNA damage

**Figure 3.**
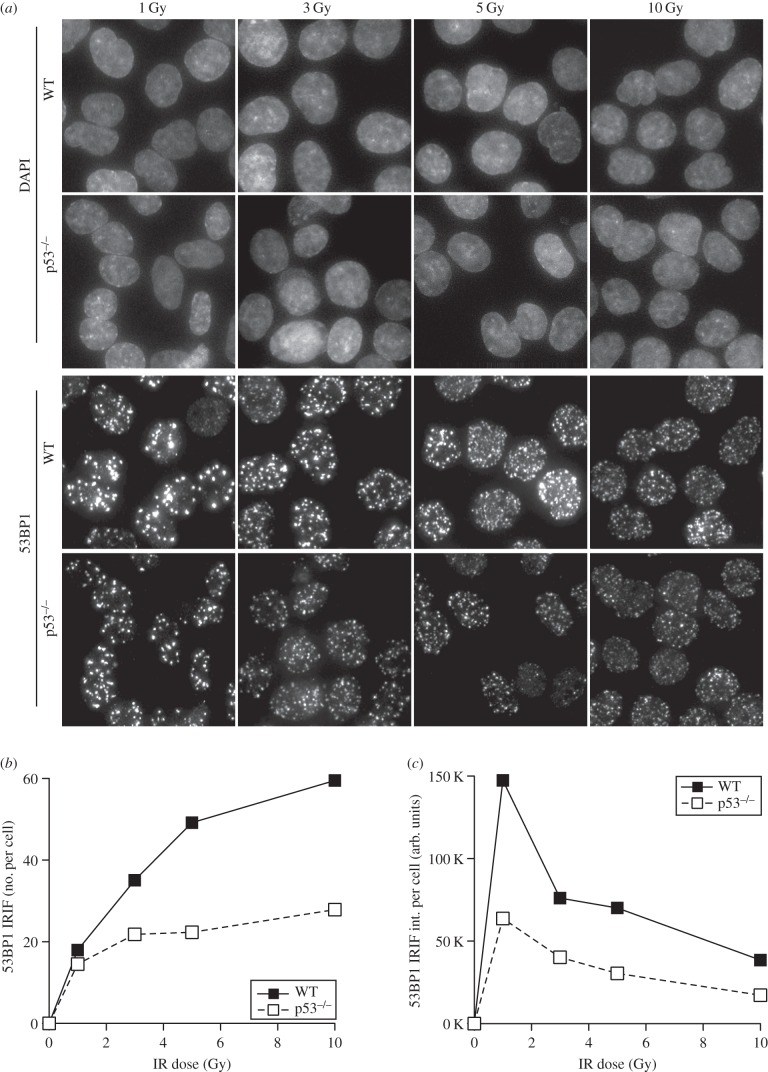
p53 promotes 53BP1 recruitment to DNA damage sites independent of IR dose. (*a*) Detection of endogenous 53BP1 by immunofluorescence in WT and p53-null HCT116 cells. Cells were irradiated, fixed after 30 min of recovery and then stained with 53Bp1 antibody. (*b*) Quantification of 53BP1 IRIF number. (*c*) Quantification of 53BP1 IRIF intensity.

The function of both p53 and 53BP1 can vary depending upon the degree of DNA damage inflicted on cells. Reparable levels of DNA damage result in p53-dependent transient arrests to cell proliferation, whereas p53 also regulates cellular senescence or apoptosis presumably after higher levels of DNA damage [42]. Similarly, 53BP1 depletion has been reported to result in a defective G2/M checkpoint at low (3 Gy) but not high (10 Gy) IR doses [43]. In addition, 53BP1 has been shown to facilitate the phosphorylation of CHK2 specifically at IR doses below 5 Gy [44]. In chicken DT40 cell lines clonogenic survival of *53Bp1* null cells displayed IR sensitivity only below 4 Gy IR [45,46]. Therefore, we followed the IR dose response of 53BP1 recruitment into foci with respect to p53 status (figure 3). Across all doses used, from low- (1 Gy) to high-dose (10 Gy) irradiation, p53-null HCT116 cells presented with both reduced numbers of detectable 53BP1 foci and weaker 53BP1 focal intensity compared with WT cells at 30 min after irradiation (figure 3*a–c*). Thus, the role of p53 in the efficient recruitment of 53BP1 into foci after ionizing radiation is dose independent.

### 2.3. p53-dependent regulation of 53BP1 IRIF is independent of the upstream mediator, MDC1

The recruitment of 53BP1 to chromatin in the proximity of DSBs results from a complex cascade of events involving the MDC1 mediator protein [47]. Specifically, in the absence of MDC1, 53BP1 IRIF formation is strongly decreased [48,49]. To determine whether p53-dependent regulation of 53BP1 IRIF occurs upstream of MDC1, we examined focal recruitment of MDC1 after IR. Depletion of p53 in HCT116 cells did not alter the recruitment of MDC1 to DNA lesions (electronic supplementary material, figure S3*a*). Neither the number nor the intensity of MDC1 IRIF was affected by the absence of p53 (electronic supplementary material, figure S3*b,c*). These data are consistent with p53-dependent regulation of 53BP1 IRIF formation and/or retention being downstream of the role of MDC1.

### 2.4. p53 regulates 53BP1 IRIF formation in a cell-cycle-dependent manner

**Figure 4.**
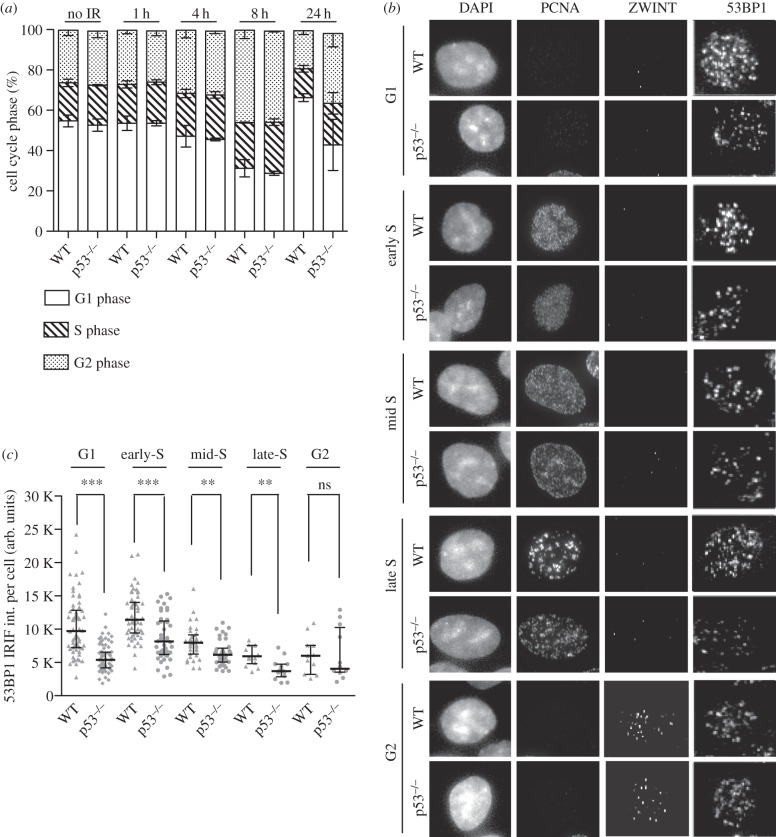
p53 regulation of 53BP1 IRIF is independent of cell cycle stage. (*a*) Quantification of the proportion of cells in each phase of the cell cycle before and after IR at the indicted times. (*b*) Detection of endogenous 53BP1 and classification with respect to cell cycle phase for each cell type analysed. S phase cells are PCNA positive, G2 phase cells are ZWINT positive, while G1 phase cells are negative for both PCNA and ZWINT. (*c*) Quantification of 53BP1 focal intensity for each cell analysed in its respective phase of the cell cycle. In total, 175 cells were scored for each cell type. Each dot represents a single cell. ***p < 0.0001, **p < 0.001, Mann–Whitney test.

It has been shown previously that upon DNA damage, 53BP1 IRIFs were larger and more intense in G0 and G1 cells, and their intensity progressively decreases during the subsequent phases of the cell cycle [24]. Less numerous and reduced intensity 53BP1 IRIF in p53-null cells are not likely to reflect a faster transit through the early phases of the cell cycle as cell cycle profiles of exponentially growing WT and p53-null HCT116 cells are very similar for at least 8 h after IR (figure 4*a*). Twenty-four hours after IR p53-null cells display a reduced proportion of cells in G1. However, as the p53-dependent defect in human 53BP1, as well as mouse 53Bp1, we observed are both well within 8 h, this defect cannot be explained by a lower proportion of G1 cells in p53-null cells.

To further assess whether cell cycle phase impacts on the role of p53 in the efficient recruitment of 53BP1 into IRIF, asynchronous WT and p53-null HCT116 cells were irradiated and 53BP1 foci formation monitored alongside PCNA and ZWINT, two cell-cycle-phase-specific markers. ZWINT is required for kinetochore assembly and can be observed as foci primarily in G2 [50]. PCNA is required for DNA replication and can be observed during the S phase in distinct focal staining patterns specific for early, mid and late stages of DNA replication. Exponentially growing cells negative for ZWINT or PCNA staining are in G1 phase. Consistent with the observations of Chapman *et al.* [24] in WT cells, we observed more efficient recruitment of 53BP1 into IRIF at early stages of the cell cycle, specifically in G1 and early S phase (figure 4*b*,*c*). The average intensity of 53BP1 foci decreased steadily, reaching a minimum in G2 phase (figure 4*c*). In the absence of p53, 53BP1 focal intensity was also observed to be at its maximum early in the cell cycle, decreasing steadily as cells progressed through S phase and into G2 as observed in WT cells. However, although the effect is most notable early in the cell cycle when 53BP1 foci are most prominent, the intensity of 53BP1 foci in *TP53^−/−^* cells is reduced relative to WT cells at all cell cycle stages (figure 4*c*). Thus, p53-dependent regulation of 53BP1 IRIF formation is largely independent of the cell cycle stage.

### 2.5. BRCA1 recruitment to DSBs is restrained by p53

There is a reciprocal relationship between 53BP1 and BRCA1 localization to DSBs [24]. 53BP1 is also known to negatively regulate HR by inhibiting DNA end resection, while BRCA1 promotes end resection [21,23,25,51]. Furthermore, the recruitment of 53BP1 to DSBs is associated with an exclusion of BRCA1 from sites of DNA damage [24,52].

**Figure 5.**
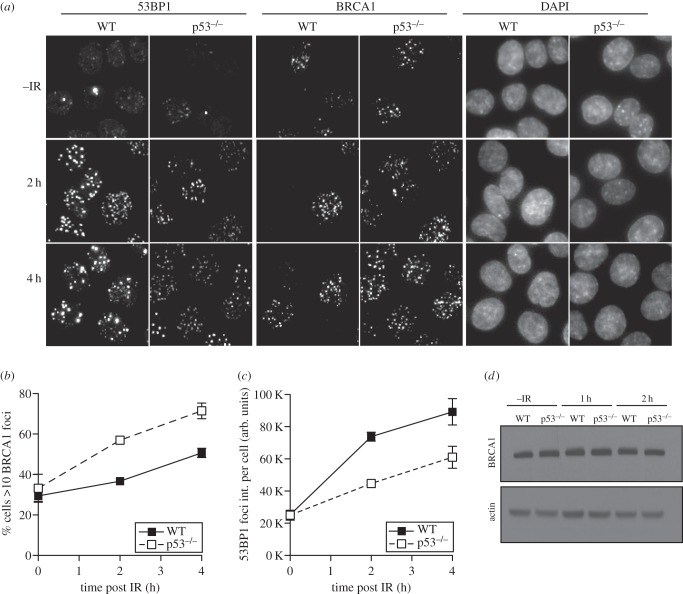
Loss of p53 results in increased recruitment of BRCA1 to DSBs. (*a*) Localization of endogenous 53BP1 and BRCA1 by immunofluorescence in HCT116 WT and p53-null cells before and after irradiation (3 Gy). (*b*) Quantification of the proportion of cells with more than 10 BRCA1 foci per cell. (*c*) Quantification of the intensity of 53BP1 foci per cell. (*d*) Western blot analysis of BRCA1 protein levels in WT and p53-null HCT116 cells at the indicated time points after IR (3 Gy).

To investigate the effect of p53 status on BRCA1 recruitment to DSBs, we evaluated BRCA1 IRIF in WT and isogenic p53-null HCT116 cells (figure 5*a*). BRCA1 also form focal structures in S phase cells [53] and, consistent with the similar cell cycle profiles of WT and p53-null cells (figure 4*a*,*b*), in both cell types about one-third of the exponentially growing unirradiated cells display BRCA1 foci (figure 5*b*). After irradiation, and as before (figure 1), while p53-null cells display reduced 53BP1 foci intensity (figure 5*a*,*c*), the proportion of cells displaying BRCA1 foci is greater in p53-null cells at both 2 and 4 h after IR (figure 5*a*,*b*). These data are consistent with p53 being a positive regulator of 53BP1 recruitment into IRIF, with 53BP1 in turn being a negative regulator of BRCA1 IRIF.

BRCA1 protein levels at late times (12–24 h) after irradiation have been reported to be p53-dependent [54]. Even though the time points after irradiation used in our study are much earlier, we nevertheless compared BRCA1 protein levels in response to IR at these early time points. We could not detect a change in BRCA1 protein levels 1 or 2 h after irradiation (figure 5*d*). Therefore, the enhanced recruitment of BRCA1 in p53-null cells is unlikely to result from altered expression of BRCA1 at early time points. Rather, it is consistent with the reduced recruitment of 53BP1, a known negative regulator of BRCA1, to DSBs at early time points.

### 2.6. p53 restrains DNA double-strand break repair via homologous recombination while promoting non-homologous end joining

**Figure 6.**
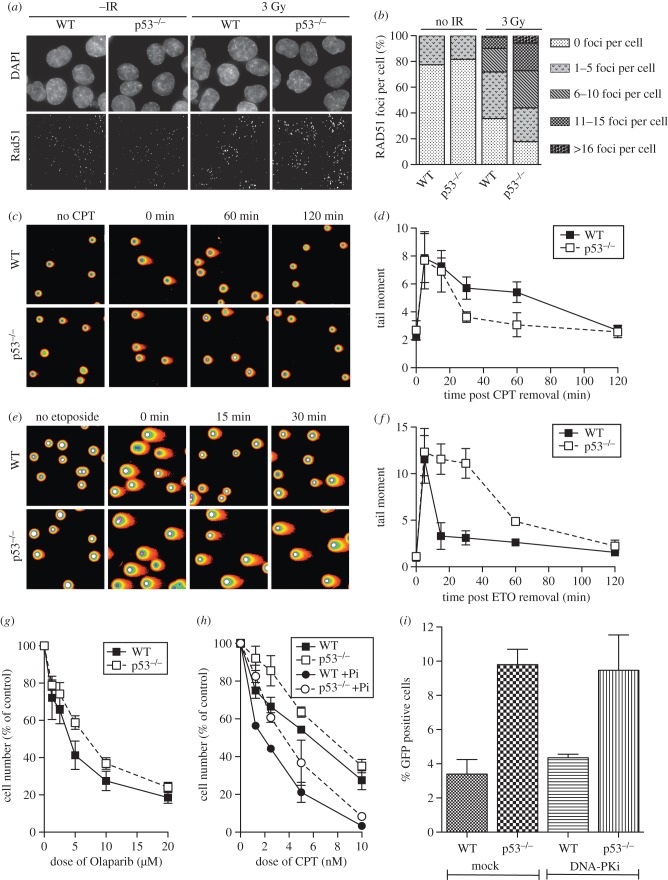
p53 inhibits HR and promotes NHEJ. (*a*) Detection of endogenous RAD51 in HCT116 WT and p53-null cells by immunofluorescence 5 h after irradiation (3 Gy). (*b*) Quantification of the numbers of RAD51 foci per cell for the indicated categories. (*c*) Representative images of a neutral comet assay (spectrum view) for WT and p53-null HCT116 cells after release from a 1 h camptothecin (CPT) treatment for the indicated times. (*d*) Quantification of CPT-induced DSBs analysed by comet assay. (*e*) Representative images of a neutral comet assay (spectrum view) for WT and p53-null HCT116 cells after release from a 1 h etoposide (ETO) treatment for the indicated times. (*f*) Quantification of ETO-induced DSBs analysed by comet assay. (*g*) Proliferation of WT and p53-null HCT116 cells in the presence of the indicated doses of Olaparib, an inhibitor of PARP. (*h*) Proliferation of WT and p53-null HCT116 cells in the presence of the indicated doses of CPT. Cells were grown in the presence of absence of 1 µM Olaparib, as indicated. (*i*) Modified GFP reporter assay for HR [55]. The percentage of live, transfected, GFP-positive cells in HCT116 WT and p53-null cells is shown. Cells were also subjected to inhibition of DNA-PK, as indicated.

Our data are consistent with a role for p53 in the promotion of 53BP1 recruitment to DSBs, which in turn restricts the accumulation of BRCA1. As BRCA1 facilitates repair of DSBs by HR, increased BRCA1 at sites of DNA damage in p53-null cells would be expected to result in increased HDR. Consistent with this reasoning, analysis of the formation of RAD51 foci revealed that the percentage of cells positive for RAD51 foci, as well as the number of RAD51 foci per cell, is increased in the absence of p53 (figure 6*a*,*b*).

To further investigate DNA repair in WT versus p53-null cells, we performed neutral comet assays to measure the resolution of DSBs induced by treatment of cells with camptothecin (CPT), an inhibitor of topoisomerase I. The collision of replication forks with CPT-induced lesions generates one-ended DSBs that are a preferential substrate for HDR [11,56]. Therefore, in asynchronously growing cell populations, it is mostly cells in S phase that present with γH2AX foci after CPT treatment. While 1 h of CPT treatment induces similar extents of DNA damage in both WT and p53-null cells, after removal of CPT, DSBs induced by this drug were progressively repaired (figure 6*c*,*d*). However, consistent with enhanced homology direct repair in the absence of p53, repair of CPT-induced lesions was completed more efficiently in p53-null cells relative to WT cells.

To investigate the role of p53 in DSB repair via the NHEJ pathway we used etoposide (ETO), an inhibitor of topoisomerase 2, reported to induce DSBs that are primarily repaired by NHEJ [12]. After 1 h of ETO treatment DSBs were assayed by neutral comet assay (figure 6*e*,*f*). The treatment resulted in similar levels of damage in both WT and p53-null cells; while repair of these ETO-induced DSBs was achieved rapidly in WT cells (within 15 min of ETO removal), in p53-null cells DSB repair was much less efficient, being still incomplete 1 h after treatment. Thus, while HDR-dependent repair of CPT-induced DSBs is more efficiently repaired in p53 defective cells, the opposite is true of NHEJ-dependent repair of ETO-induced DSBs as these lesions are less efficiently repaired in p53 defective cells.

To further assess HDR in WT and p53-null cells, we examined cell proliferation in the presence of Olaparib, an inhibitor of PARP inhibitor (figure 6*g*). PARP is required for efficient repair of single-stranded breaks (SSBs) and its inhibition results in conversion of SSBs into DSBs that are primarily repaired via HDR [57,58]. Thus, sensitivity to PARP inhibition can be used as a read-out for defective HDR. *TP53^−/−^* cells proliferated more rapidly in the presence of a range of Olaparib concentrations than did WT cells, as would be expected for cells with greater capacity for HDR. We also combined low-level PARP inhibition with increasing concentration of CPT (figure 6*h*). Consistent with previous reports using a different PARP inhibitor, KU58948 [59], p53-null cells displayed enhanced proliferation relative to WT cells both in the absence or presence of Olaparib.

Finally, in order to directly investigate the efficiency of HDR, we transiently transfected pDR-GFP, a GFP reporter construct specific for HDR [55], into WT and p53-null cell lines (figure 6*i*). Relative to WT, an increased efficiency of HDR in p53-null cells was measured. Interestingly, the efficiency of HDR in WT cells could be stimulated, as expected, by inhibition of the competing NHEJ pathway (using NU7026, an inhibitor of DNA-PK). However, a further increase in HDR efficiency was not observed when p53-null cells were treated with NU7026, suggesting that HDR has reached its full capacity under these conditions.

Our data are consistent with p53 reciprocally regulating the two major pathways of DSB repair. Specifically, p53 is a positive regulator of NHEJ but a negative regulator of HDR, suggesting that p53 is required for fine-tuning the balance between these two competing pathways of DSB repair.

## 3. Discussion

The mechanism behind 53BP1 recruitment to DSBs is still not fully characterized. Earlier studies established roles for both the 53BP1 oligomerization and Tudor domains for its recruitment into foci after ionizing radiation [60]. A more recent study has established that stable retention of 53BP1 at chromatin surrounding DSBs requires a newly described ubiquitin-binding domain and RNF8/RNF168-dependent ubiquitination [31]. With respect to the 53BP1 Tudor domain, two distinct histone modifications, H3K79me2 and H4K20me2, have been reported to be required for 53BP1 recruitment to sites of DNA damage [34,61]. While the relationship between H3K79me2 and 53BP1 recruitment to chromatin in the proximity of DSBs remains unclear, lack of H4K20me2 is consistently associated with a defect in 53BP1 recruitment to DSBs. However, the extent of the 53BP1 recruitment defect reported in H4K20me2-deficient cells varies between studies. In HeLa cells, it has been shown that lack of H4K20me2 abrogates 53BP1 foci formation for at least an hour following IR treatment [34–36], while in MEFs depleted for H4K20me2, 53BP1 foci formation was merely delayed for the first 5 min post-IR treatment [37]. Interestingly, one noteworthy difference between the two cell lines used in these studies is their p53 status, with MEFs being WT for p53 while HeLa cells are defective for p53 function.

A complex between 53BP1 and p53 was originally shown to be associated with upregulation of p53 transcriptional activity [9]. More recently, a newly identified post-transcriptional modification of p53, p53K382me2, has been shown to have affinity for the 53BP1 tandem Tudor domain and is induced upon DNA damage [38,40]. These authors suggested that the presence of 53BP1 at DSBs might help to recruit and stabilize p53 at DSBs in order to regulate p53 functions that are independent of its known transcriptional transactivation activity.

In this study, we observed that the formation of 53BP1 foci at DSBs is abrogated in the absence of p53 in both human and mouse cells. This p53-dependent defect in 53BP1 ionizing radiation-induced foci (IRIF) was IR dose-independent, while 53BP1 protein levels were unaffected by the absence of p53, suggesting a regulatory role for p53 in recruitment of 53BP1 into IRIF. We found that loss of p53 resulted in defective accumulation of 53BP1 into IR-induced foci that was both immediate and persistent in both human and mouse cells. This contrasts with a report showing that MEFs defective for the histone H4K20me2 modification but WT for p53 displayed only a modest defect, restricted to just the first 5 min after irradiation, in 53BP1 recruitment into IRIF [37].

Although many details remain to be deciphered, our data are consistent with a model in which the recruitment of 53BP1 to DSBs involves complex steps that require both direct interactions with histones, as well as interactions with non-histone proteins. The initial histone-dependent process involves dynamic interactions between the Tudor domain of 53BP1 and a constitutive chromatin mark, H4K20me2, that may be more easily accessed around DSBs [62]. This initial histone-dependent interaction is then stabilized via another histone-dependent interaction between the recently described 53BP1 ubiquitin-binding domain and RNF168-dependent ubiquitination of H2A-type histones, an interaction that also requires 53BP1 oligomerization [31]. Finally, 53BP1 retention at DSBs also requires the damage-inducible γH2AX modification [49]. Our data suggest that in addition to these directly histone-dependent processes, p53 also plays a role in the accumulation and stabilization of 53BP1 at DSBs.

The defect in p53-dependent 53BP1 accumulation at DSBs is prevalent in G1 and early S phase, and then progressively diminishes in mid and late S phase, becoming minimal in G2. The G1 and early S phases correspond to the predominant phase of the cell cycle for the NHEJ repair. Although only relevant to DSBs that do not remain in close proximity, one reported mechanism by which 53BP1 promotes NHEJ is enhancing the mobility of broken chromatids [22]. Of more relevance to all DSBs, 53BP1 is also known to decrease HDR by downregulating DNA end resection [21–23]. Indeed, this initial step in the HDR pathway is dependent upon the balance between 53BP1 and the BRCA1 protein that promotes the resection of DNA ends [21]. It has been shown using super-resolution microscopy that recruitment of BRCA1 into IRIFs correlates with exclusion of 53BP1 away from the focal core and towards its periphery [24]. Consistent with this observation, we observed that the abrogation of 53BP1 IRIF in the absence of p53 is accompanied by enhanced BRCA1 foci formation, which in turn resulted in increased formation of RAD51 IRIF.

We confirmed a role for p53 in regulating DSB repair by demonstrating that DSB repair of CPT-induced lesions, which are preferentially repaired by HR, were more efficiently repaired in the absence of p53. Correspondingly, p53-deficient cells are more efficient at repairing ETO-induced lesions that are preferentially repaired by NHEJ. Additionally, they are less sensitive to PARP inhibition and exhibit elevated levels of HDR. Altogether, our results suggest a new function for p53 as a regulator of the balance between HDR and NHEJ through its stimulation of efficient recruitment of 53BP1 to sites of DNA damage. The requirement for p53 in the efficient recruitment of 53BP1 into IRIF is most striking in G1 and early S phase of the cell cycle. As reduced 53BP1 recruitment is accompanied by a reciprocal increase in BRCA1 recruitment it is likely that inappropriate upregulation of HDR, despite being an error-free pathway, could be threatening for genome integrity. In G0 and G1 cells, the absence of a homologous sister chromatid could result in the loss or even rearrangement of genetic information.

Discovered over 35 years ago and regarded as a ‘guardian of the genome’, p53 is one of the most studied yet functionally complex proteins in biochemistry. With respect to its roles in the DDR, these are largely as ‘effectors’ of transient cell cycle delays and cellular fate. Our results highlight a new role for p53 in ‘mediating’ early events of the DDR important for regulating the balance between DSB repair pathways.

## 4. Material and methods

### 4.1. Cell culture and transfection

HCT116 and p53-null (*TP53^−/−^*) derivative cells were supplied by B. Vogelstein [63]. HCT116 cells were grown in DMEM media with 10% FBS (Lonza) and 1% PenStrep (Sigma). MEFs and *Trp53-*null (*Trp53^−/−^*) derivative cells were a gift from S. Jones (University of Massachusetts). MEFs were grown in DMEM media supplemented with 15% FBS and 1% PenStrep.

### 4.2. Cell extracts and western blotting

Harvested cells were washed in cold PBS, resuspended in sample buffer (5 µl 5 × SB per 2 × 10^5^ cells), lysed by heating at 95°C for 10 min, sonicated (40% amplitude, 10 s, Branson 250 Sonicator) and heated at 95°C for a further 10 min. Lysed extracts were subject to SDS-PAGE and transferred to nitrocellulose membranes by electroblotting. The membranes were blocked with 4% milk, incubated overnight with 1° antibody as indicated, washed and incubated with HRP-coupled secondary antibodies as relevant. Antibodies used for western blotting were anti-53BP1 (Novus #NB100-904, 1/1000), anti-γH2AX (Millipore #05-636, 1/2000), anti-BRCA1 (Santa Cruz, D-9 #sc6954, 1/500), anti-p53 (Cell Signalling, #9282, 1/1000), anti-p53 DO1 (Santa Cruz, #sc-126, 1/1000) and anti-ATR (Santa Cruz, #sc-1887, 1/2000).

### 4.3. Immunofluorescence and microscopy

Human HCT116 cells or mouse MEFs, either WT or null for p53, were fixed with 4% PFA and permeabilized with 0.125% of Triton-X100. After briefly blocking in 4% BSA, cells were incubated for 1 h at 37°C with 1° antibody, washed and incubated for 1 h at 37°C with 2° antibody. Slides were mounted using Vectashield mounting media with DAPI (Vector Laboratories). The following antibodies were used for Immunofluorescence staining: anti-53BP1 (Novus Biological, #NB100-904, 1/400), anti-γH2AX (Millipore, #05-636, 1/200), anti-BRCA1 (Santa Cruz, #sc6954, 1/500), anti-PCNA (Kevin Sullivan, CCB), anti-ZWINT (Kevin Sullivan, CCB) and anti-RAD51 (Abcam, #ab63801, 1/200). Microscopy imaging was performed on a Deltavision microscope using Softworx software (Applied Precision, Issaquah). Z-stacks (0.5 µm) were collected, deconvolved and projected. Quantification of foci was performed using Image-Pro Analyser software (MediaCybernetics).

### 4.4. Cell cycle analysis

HCT116 WT or *TP53*^−/−^ cells were plated at 2 × 10^5^ cell per 35 mm dish and grown for 24 h prior to treatment. Cells were then treated with 25uM BRDU for 1 h, washed with PBS and fresh media was added. Cells were then γ-irradiated (3 Gy) with a caesium-137 source (Mainance, UK), harvested at the indicated times, fixed in 70% ice-cold ethanol, washed with PBS and the DNA was denatured using 2N HCl for 10 min before being stained with anti-BRDU antibody (B-D, Ca #347580) for 1 h and anti-mouse secondary for 1 h. Cells were then stained with propidium iodide solution (PI) (40 µg ml^−1^ of PI (Sigma) and 250 µl ml^−1^ of RNAse A (Qiagen) in PBS) for 30 min in the dark. The analysis was performed using BD FACSCantoII and BD FACSDiva software (BD Biosciences).

### 4.5. Comet assay

Cells were treated with 1.25 µM CPT or 50 µM ETO for 1 h, washed with PBS then collected at the indicated time of recovery. The neutral comet assay method was adapted from the manufacturer's instructions (Trevigen). Cells were harvested, combined with LMA agarose (Trevigen) at a concentration of 1 × 10^5^ cells ml^−1^ and loaded on polylysine slides. The slides were incubated at 4°C in the dark for 30 min to allow the agarose to set. Cell lysis was performed by placing the slides in ice-cold lysis buffer overnight and neutralized in neutral electrophoresis buffer for 30 min. Slides were then place in an electrophoresis chamber and run for 1 h at 24 V corresponding to 1 V cm^−1^ between electrodes. Cells trapped in agarose were treated with DNA precipitation buffer and washed with 70% ethanol. Slides were allowed to dry at 37^°^C before staining with SyBR-green and visualized by microscopy. COMET analysis was performed using the software CometScore from Tritek Corporation.

### 4.6. Cell proliferation assays

HCT116 WT or *TP53*^−/−^ cells were plated at 2 × 10^5^ cell per 35 mm dish 24 h prior to treatment. For Olaparib treatment, new media containing the drug at the indicated concentrations was added to the cells and the cells were cultured for 48 h. After 48 h of drug treatment, cells were trypsinized and re-plated in drug-free media onto two 35 mm dishes to ensure optimal growth conditions. After culturing for a further 48 h in drug-free media, cells were harvested by trypsinization and counted. For campthotecin (CPT) treatment, fresh media containing the drug at the indicated concentrations was added to the cells. Addition of PARP inhibitor (1uM Olaparib) was as indicated and the cells were grown for a further 48 h. Cells were then trypsinized and re-plated in drug-free media onto two 35 mm dishes to ensure optimal growth conditions for a further 48 h after which cells were harvested by trypsinization and counted.

### 4.7. GFP reporter assays

HCT116 WT or *TP53^−/−^* cells were plated at 1 × 10^6^ cells per 35 mm dish 24 h prior to transfection. Cells were co-transfected with 1 µg pCerulean-N1 (Addgene #54742), expressing Cerulean Fluorescent Protein to identify transfected cells, 5 µg pDR-GFP [55] and 5 µg pCBA-I-SceI [55] using Lipofectamine (Invitrogen) and cultured as normal. The DNA-PK inhibitor, NU7026 (Tocris Biosciences), was used as a control as the level of HR increases upon inhibition of the competing NHEJ pathway. 48 h after co-transfection, cells were trypsinized and resuspended in 500ul PBS containing 40 nM TO-PRO-3 iodide (Life Technologies, #T3605) to identify live cells. FACS analysis was carried out using BD FACSCantoII and BD FACSDiva software. Briefly, cells were gated as follows: live cells (ToPro3 negative), singlet cells (FSC-A verses FSC-H), transfected cells (Cerulean positive). The percentage of GFP-positive cells was derived from the live, transfected, single-cell population.

## Supplementary Material

Moureau et al. All Supplemental Material
