# Supplementary material for: A role for the p53 tumour suppressor in regulating the balance between homologous recombination and non-homologous end joining
Source: Open Biol. 2016 Sep 21;6(9):160225. doi: 10.1098/rsob.160225 (PMC5043586; doi:10.1098/rsob.160225)
Supplement: Moureau et al. All Supplemental Material [file rsob160225supp1.pdf]

A role for the p53 tumour suppressor in regulating the balance between homologous recombination and non-homologous end-joining. *Moureau et al.* ; Open Biology

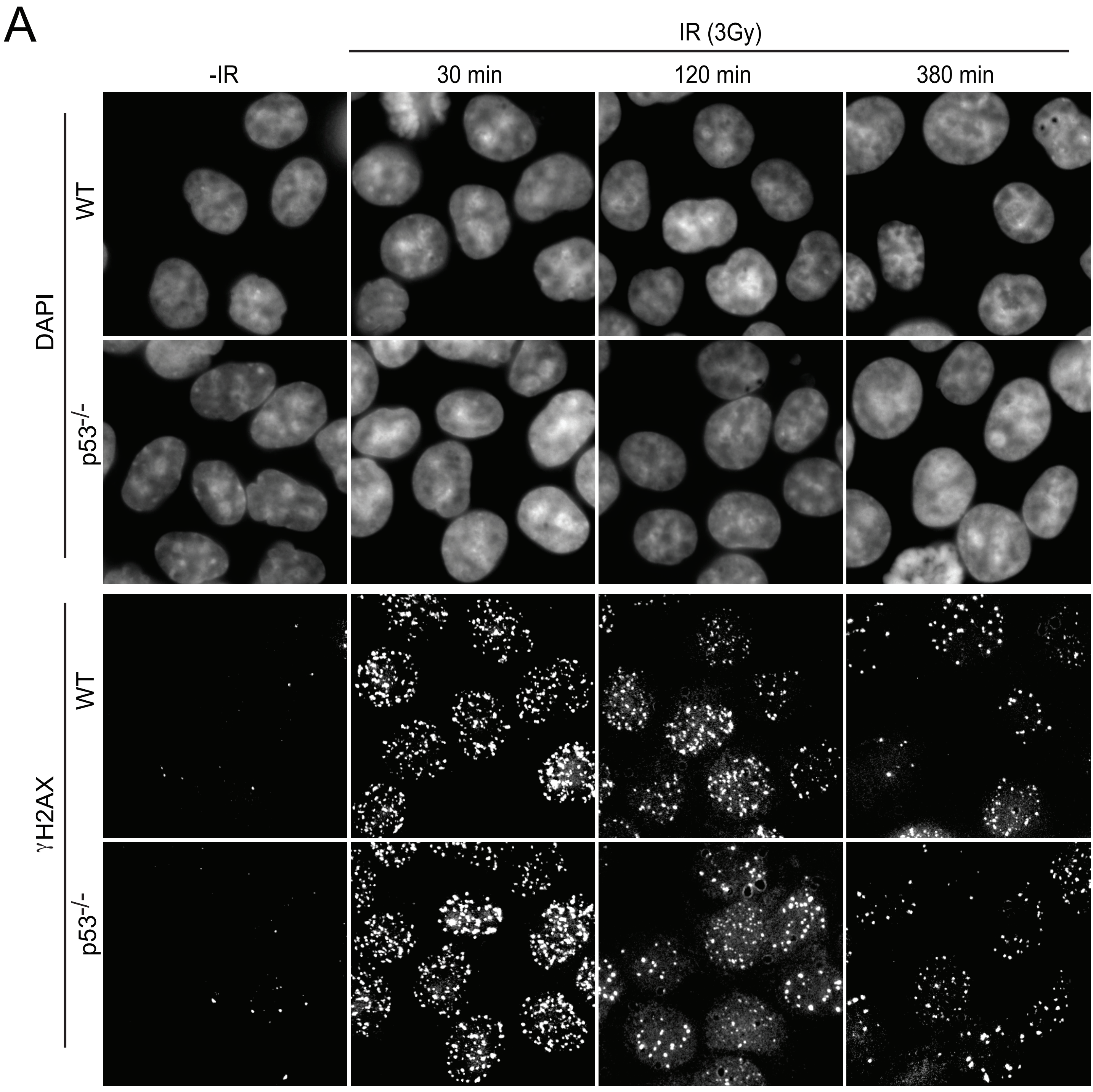

Supplemental Figure 1: p53 does not affect the γH2AX modification at sites of DNA damage in human HCT116 cells. (A) Detection of γH2AX foci by immunofluorescence. HCT116 cells were irradiated with 3Gy, fixed and then stained with γH2AX antibody.

A role for the p53 tumour suppressor in regulating the balance between homologous recombination and non-homologous end-joining. *Moureau et al.* ; Open Biology

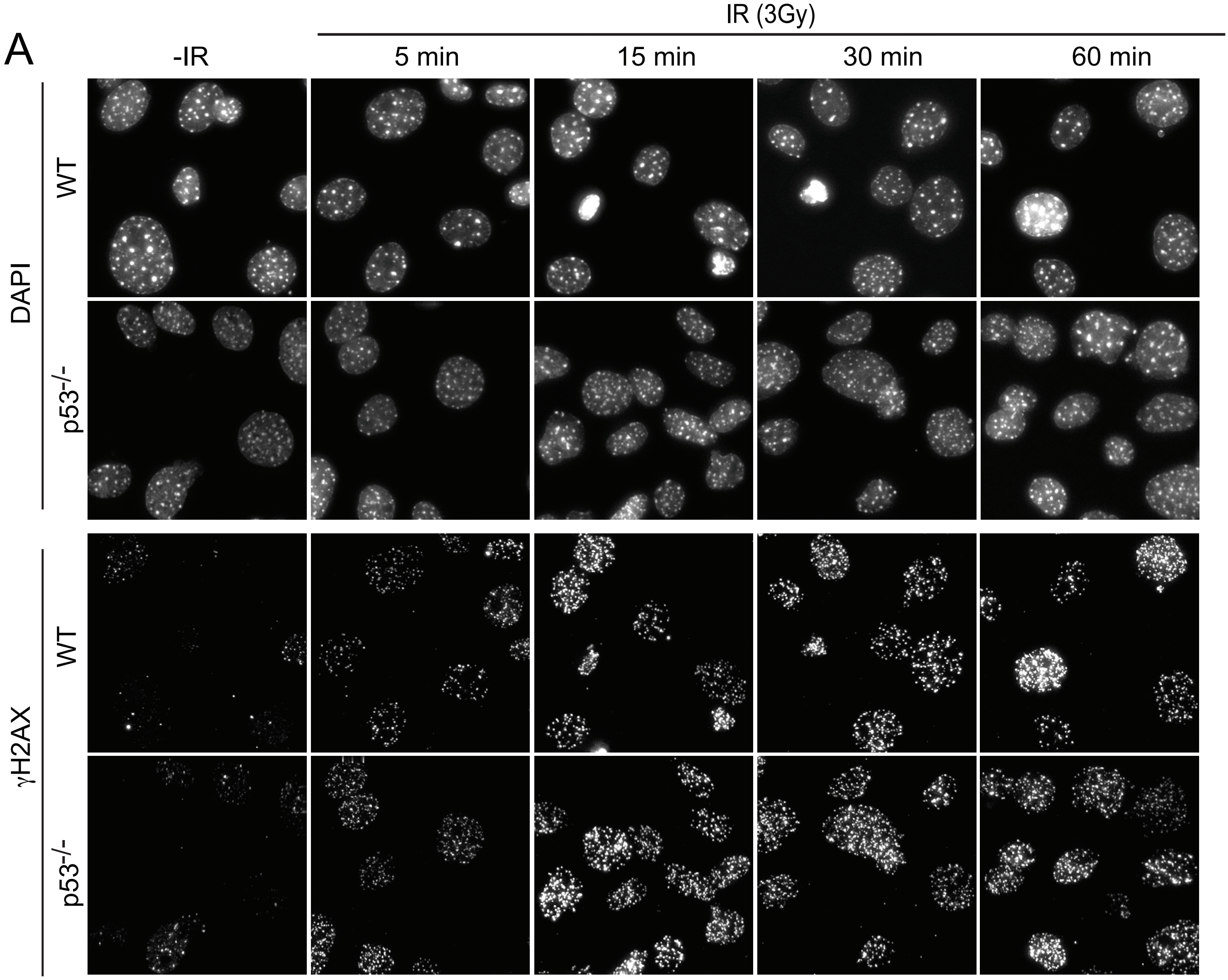

Supplemental Figure 2: p53 does not affect the γH2AX modification at sites of DNA damage in MEFs. (A) Detection of γH2AX foci by immunofluorescence in early passage MEFs.

A role for the p53 tumour suppressor in regulating the balance between homologous recombination and non-homologous end-joining. *Moureau et al.* ; Open Biology

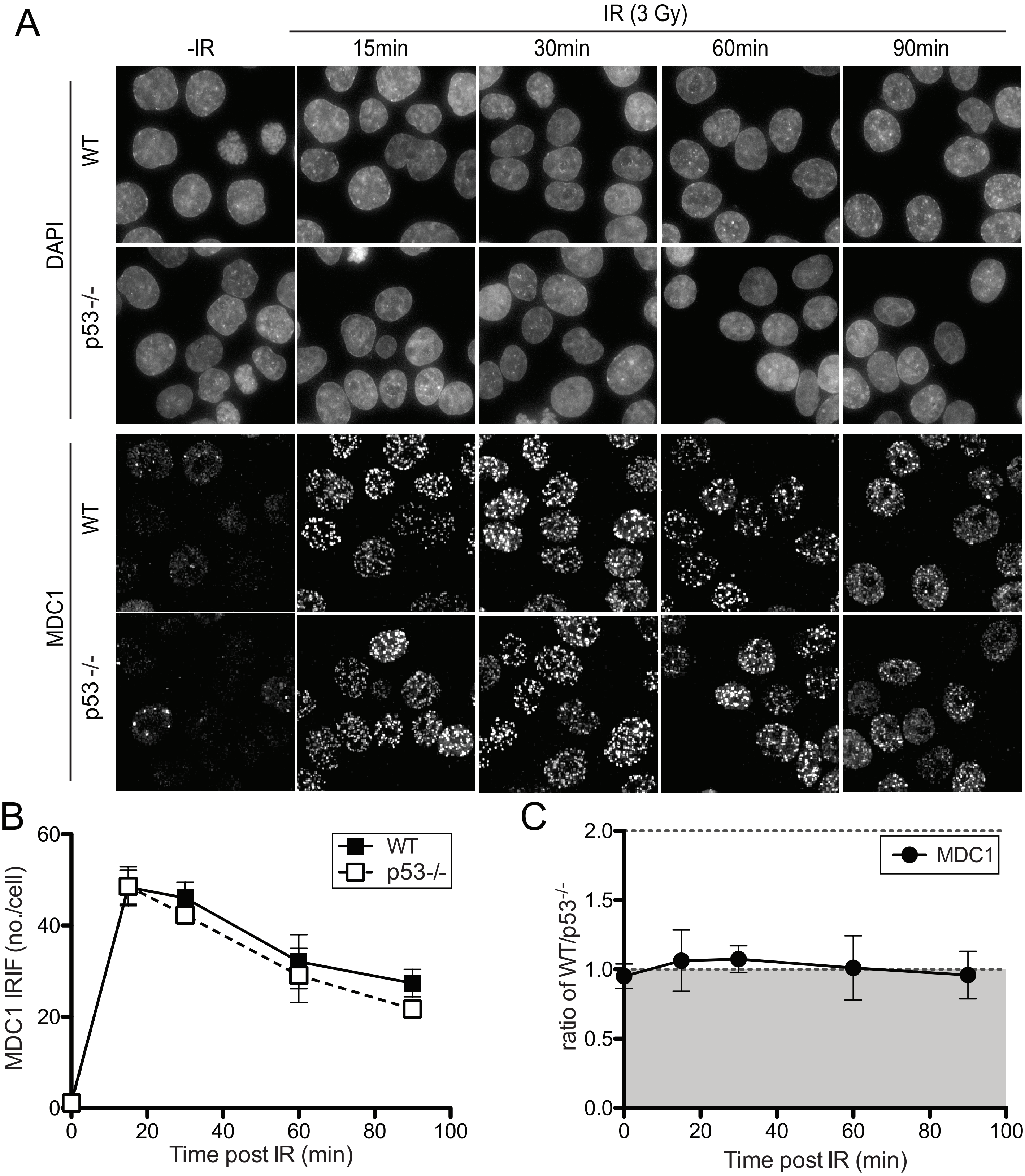

Supplemental Figure 3: MDC1 recruitment to DSBs is p53 independent. (A) Detection of endogenous MDC1 by immunofluorescence in WT and p53 null HCT116 cells. (B) Quantification of the number of MDC1 foci. (C) The ratio of MDC1 focal intensity between WT and p53 null cells.
